# Sustainable Production of *N-*methylphenylalanine by Reductive Methylamination of Phenylpyruvate Using Engineered *Corynebacterium glutamicum*

**DOI:** 10.3390/microorganisms9040824

**Published:** 2021-04-13

**Authors:** Anastasia Kerbs, Melanie Mindt, Lynn Schwardmann, Volker F. Wendisch

**Affiliations:** 1 Genetics of Prokaryotes, Faculty of Biology and CeBiTec, Bielefeld University, 33615 Bielefeld, Germany; anastasia.kerbs@uni-bielefeld.de (A.K.); l.schwardmann@uni-bielefeld.de (L.S.); 2 BU Bioscience, Wagenigen University and Research, 6700AA Wageningen, The Netherlands; melanie.mindt@wur.nl

**Keywords:** *N*-functionalized amines, sustainable production of *N*-methylphenylalanine, *Corynebacterium glutamicum*, metabolic engineering, DpkA

## Abstract

*N-*alkylated amino acids occur widely in nature and can also be found in bioactive secondary metabolites such as the glycopeptide antibiotic vancomycin and the immunosuppressant cyclosporine A. To meet the demand for *N-*alkylated amino acids, they are currently produced chemically; however, these approaches often lack enantiopurity, show low product yields and require toxic reagents. Fermentative routes to *N-*alkylated amino acids like *N-*methyl-l-alanine or *N-*methylantranilate, a precursor of acridone alkaloids, have been established using engineered *Corynebacterium glutamicum*, which has been used for the industrial production of amino acids for decades. Here, we describe metabolic engineering of *C. glutamicum* for de novo production of *N-*methylphenylalanine based on reductive methylamination of phenylpyruvate. *Pseudomonas putida* Δ-1-piperideine-2-carboxylate reductase DpkA containing the amino acid exchanges P262A and M141L showed comparable catalytic efficiencies with phenylpyruvate and pyruvate, whereas the wild-type enzyme preferred the latter substrate over the former. Deletion of the anthranilate synthase genes *trpEG* and of the genes encoding branched-chain amino acid aminotransferase IlvE and phenylalanine aminotransferase AroT in a strain engineered to overproduce anthranilate abolished biosynthesis of l-tryptophan and l-phenylalanine to accumulate phenylpyruvate. Upon heterologous expression of *DpkA*^P262A,M141L^, *N-*methylphenylalanine production resulted upon addition of monomethylamine to the medium. In glucose-based minimal medium, an *N-*methylphenylalanine titer of 0.73 ± 0.05 g L^−1^, a volumetric productivity of 0.01 g L^−1^ h^−1^ and a yield of 0.052 g g^−1^ glucose were reached. When xylose isomerase gene *xylA* from *Xanthomonas campestris* and the endogenous xylulokinase gene *xylB* were expressed in addition, xylose as sole carbon source supported production of *N-*methylphenylalanine to a titer of 0.6 ± 0.04 g L^−1^ with a volumetric productivity of 0.008 g L^−1^ h^−1^ and a yield of 0.05 g g^−1^ xylose. Thus, a fermentative route to sustainable production of *N-*methylphenylalanine by recombinant *C. glutamicum* has been established.

## 1. Introduction

*N-*methylated amino acids are found in bacteria and eukaryotes. These non-proteinogenic amino acids occur in peptides or as free monomers. As an example of the latter, the *N*^5^-ethylated l-glutamine derivate l-theanine is found in green tea leaves and is responsible for its characteristic umami taste [1,2]. As *S*-adenosylmethionine (SAM) is the methyl donor for almost all cellular methylation reactions occurring in nature, the level of SAM must be regulated in response to metabolic changes [3]. The glycine *N-*methyltransferase is methylating glycine in a SAM-dependent manner in mammalian cells to generate sarcosine, which plays a critical role in SAM homeostasis [4]. The tri-*N-*methylated glycine derivative glycine betaine is used pharmaceutically as adjunctive treatment for homocystinuria in its anhydrous form. Additionally, it has several other benefits to human health as it is acting like an attenuator of liver injury [5]. Additionally, *N-*alkylated amino acids occur as intermediates of metabolic pathways such as *N-*methylglutamate in the C1 assimilation of monomethylamine by methylotrophic bacteria such *Methyloversatilis universalis* [6].

A number of bioactive peptides contain *N-*methylated amino acids such as the anticancer cytostatic agent actinomycin D, the glycopeptide antibiotic vancomycin and the immunosuppressant cyclosporine A [7,8]. In peptidomimetics (synthetic peptides) a proteinogenic amino acid may be substituted for its *N-*methylated derivate. As a consequence of this substitution, pharmacological properties such as stability against proteolytic degradation, receptor selectivity [9] and membrane permeability [10] can be enhanced. For example, the *N-*methylated peptidomimetic of the antitumor drug Somatostatin showed a higher bioavailability due to a higher membrane permeability coefficient [11]. The natural peptide β-amyloid, causing Alzheimer’s disease, is forming fibrillar aggregates and is highly toxic. The so called “β-sheet breakers” can prevent fibrillogenesis and revert amyloid formation, which lowers the toxicity of Alzheimer’s peptides [12,13]. The *N-*methylated β-amyloid derivates are preventing aggregation and inhibiting the resulting toxicity of the native peptide [14,15,16]. *N-*methylphenylalanine (NMePhe) is the *N-*terminal amino acid in pilis of most bacterial pathogens possessing type IV pili [17]. NMePhe-rich peptides find application as blood–brain barrier shuttles [18]. Taken together, the production of free *N-*methylated amino acids as well as peptides with *N-*methylated amino acids gains increasing interest in the pharmaceuticals and chemical industries.

Chemical synthesis of *N-*methylated amino acids via several routes is known, including ring opening of 5-oxazolidinones, direct methylation and reductive amination [8,19,20]. However, chemical synthesis of *N-*methylated amino acids often uses toxic reagents and is limited by incomplete enantiopurity, low product yields or over-methylation [8,19]. As a greener alternative, fermentative production of *N-*methylated amino acids using methyltransferases, dehydrogenases and reductases has been developed in recent years [21]. Metabolic engineering strategies have been established for reductive alkylamination of 2-oxoacids with monomethylamine as methyl donor. One strategy is making use of the C1 assimilation pathway of *Methylobacterium extorquens* to enable glycerol-based production of *N-*methylglutamate in *Pseudomonas putida* [22]. Further, the side activity of the imine reductase DpkA from *P. putida* was used for production of *N-*methylated amino acids. DpkA natively catalyzes the reduction of Δ-1-piperideine-2-carboxylate to l-pipecolic acid in d-lysine catabolism of *P. putida* [23]. Additionally, this enzyme is able to catalyze the reductive methylamination of 2-oxoacids. Expression of *dpkA* in *Corynebacterium glutamicum* engineered to overproduce the respective 2-oxoacid precursor [24,25,26] allowed fermentative production of sarcosine [27], *N-*methyl- l-alanine [28] and *N-*ethylglycine [29] upon addition of either monomethylamine or monoethylamine to the growth medium. *C. glutamicum* is a logical choice for production of non-proteinogenic amino acids such as pipecolic acid [30,31], and trans-hydroxyproline [32,33], aromatic amino acids like 7-chloro-or 7-bromo-tryptophan [34,35] or the *N-*alkylated amino acid *N*-methylanthranilate [36] since it is used for more than 50 years for safe production of the food and feed amino acids l-glutamate [37] and l-lysine [38] at the million-ton scale [39].

**Figure 1:**
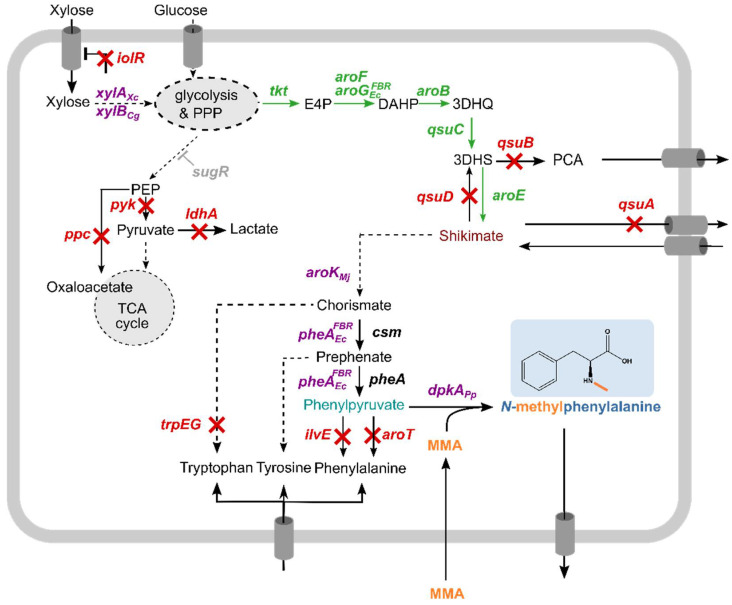
Simplified scheme of NMePhe biosynthesis. Single reactions are shown as continuous arrows, dashed arrows indicate multiple reactions. Genes and arrows depicted in green indicate genome-based overexpression, whereas purple genes indicate vector-based expression. Crossed arrows and red names indicate gene deletion. Monomethylamine as methyl donor is marked in orange. Grey *sugR* indicates reversion of deleted *sugR* back to wild type s*ugR*. PEP, phosphoenolpyruvate; TCA, tricarboxylic acid; PPP, pentose phosphate pathway; E4P, erythrose-4-phosphate; DAHP, 3-deoxy-d-arabinoheptulosonate-7-phosphate; 3DHQ, 3-dehydroquinate; 3DHS, 3-dehydroshikimic acid; PCA, protocatechuic acid; *iolR*, transcriptional regulator; *sugR*, transcriptional regulator; *xylA*, xylose isomerase from *Xanthomonas campestris*; *xylB*, *xylulokinase*; *ppc*, phosphoenolpyruvate carboxylase; *ldhA*, lactate dehydrogenase; *pyk*, pyruvate kinase; *tkt*, transketolase; *aroF*, DAHP synthase; *aroG*^FBR^, feedback-resistant DAHP synthase from *Escherichia coli*; *aroB*, 3-dehydroquinate synthase; *qsuC*, 3-dehydroquinate dehydratase; *qsuB*, 3-dehydroshikimate dehydratase; *qsuD*, shikimate dehydrogenase; *aroE*, shikimate dehydrogenase; *qsuA*, putative shikimate importer; *aroK*, shikimate kinase from *Methanococcus jannaschii*; *csm*, chorismate mutase; *pheA*^FBR^, feedback-resistant chorismate mutase/prephenate dehydratase from *Escherichia coli*; *aroT*, aminotransferase; *ilvE*, branched-chain aminotransferase; *dpkA*, imine reductase from *Pseudomonas putida*.

Here, we describe the first route to fermentative production of the secondary metabolite NMePhe, a constituent of peptides enabling passive blood–brain barrier permeation. A strain accumulating the 2-oxoacid phenylpyruvate was constructed (Figure 1). The amino acid exchanges P262A and M141L introduced into DpkA were shown to shift substrate preference such that catalytic efficiencies with phenylpyruvate and with the central intermediate of carbon metabolism pyruvate were comparable. Heterologous expression of *dpkA*^P262A,M141L^ in the constructed phenylpyruvate overproducing strain enabled fermentative production of *N-*methylphenylalanine de novo using mineral salts medium with monomethylamine as alkylamine substrate of DpkA and either glucose or xylose as carbon source.

## 2. Materials and Methods

### 2.1. Bacterial Strains and Growth Conditions

The strains and plasmids used in this work are listed in Table 1 and Table S1, respectively. *E. coli* DH5α was used for plasmid construction and was cultivated in lysogeny broth (LB) at 37 °C (180 rpm). As a host organism for phenylpyruvate and *N-*methylphenylalanine production the *C. glutamicum* chassis strain C1* strain was used. Pre-cultures of *C. glutamicum* were inoculated from a fresh LB agar plate and cultivated in brain heart Infusion (BHI) medium at 30 °C in baffled shake flasks on a rotary shaker (120 rpm). If necessary, spectinomycin (100 µg mL^−1^), tetracycline (5 µg mL^−1^) and kanamycin (25 µg m L^−1^) were added to the medium. For growth and production experiments cells were harvested (3200× *g*, 7 min) and washed once in TN buffer pH 6.3 (50 mM Tris-HCL, 50 mM NaCl) before inoculation to an optical density at 600 nm (OD_600_) to 1 in CGXII minimal medium with standard or reduced nitrogen source to either 50 % (2.5 instead of 5 g L^−1^ urea and 10 instead of 20 g L^−1^ ammonium sulfate) or 10% (0.5 instead of 5 g L^−1^ urea and 2 instead of 20 g L^−1^ ammonium sulfate). As sole carbon source 40 or 20 g L^−1^ glucose was used. The growth was followed by measuring OD_600_ using V-1200 spectrophotometer (VWR, Radmor, PA, USA). For induction of gene expression from expression vectors pEKEx3, pVWEx1 and pEC-XT99A 1 mM isopropyl-β-D-1-thiogalactopyranoside (IPTG) in final concentration was added to the medium. Additionally, the aromatic amino acids l-tryptophan (0.2 mM) and l-phenylalanine (0.8 mM) and the branched chain amino acids l-isoleucine and l-leucine (0.8 mM each) were added to the culture for auxotrophic *C. glutamicum* strains. Monomethylamine (MMA) was added as methyl donor for DpkA in concentrations from 0.1 to 0.35 M as indicated.

Growth experiments were performed in 24-well Duetz-plates. Glucose (20 and 40 g L^−1^) and xylose (5 and 12 g L^−1^) were tested as sole carbon sources. The shaking frequency was adjusted to 220 rpm for sufficient aeration. In Duetz-plates, *C. glutamicum* cells were inoculated in a total volume of 3 mL CGXII minimal medium to OD_600_ of 0.5 or 1.

To investigate whether *N-*methylphenylalanine can be used as sole carbon or nitrogen source the BioLector microcultivation system (m2p-labs, Aachen, Germany) was used. The shaking frequency was set to 1200 rpm and 48-well flower plate wells with cultivation volumes of 1 mL were used and growth was followed by backscattered light at 620 nm and a signal gain of 35.

### 2.2. Molecular Genetic Techniques and Strain Construction

Standard molecular genetic techniques were performed as described elsewhere [43]. Transformation of competent *E. coli* (prepared by RbCl method) was performed by heat shock at 42 °C [41], whereas transformation of competent *C. glutamicum* cells was carried out by electroporation [44]. PCR fragments were amplified using the respective primer (Table S1) with ALLin^TM^ HiFi DNA Polymerase according to the manufacturer (highQu GmbH, Kraichtal, Germany). The PCR products were assembled via Gibson Assembly with pEKEx3, pEC-XT99A or pVWEx1 linearized by BamHI restriction. For construction of the feedback resistant variant of the bifunctional enzyme chorismate mutase/prephenate dehydratase (encoded by *pheA*) first the gene *pheA* was amplified from genomic *E. coli* MG1655 DNA and cloned into pEKEx3 vector. The amino acid exchange causing a feedback resistant variant was performed using pEKEx3-*pheA* via site directed mutagenesis (SDM) yielding pEKEx3-*pheA*^FBR^. Used primer pairs are listed in Table S1. The plasmid pK19*mobsacB*-ΔNCgl2922::*P_tuf_-aroK^m^* was used as a template for *aroK* amplification. A three fragment Gibson Assembly (linearized pEKEx3 plasmid, *pheA*^FBR^, *aroK* with an optimized artificial RBS using the RBS calculator from Salis laboratory at Penn State University in front of *aroK*) was performed to yield plasmid pEKEx3-*pheA*^FBR^-*aroK*.

Chromosomal deletions and integrations were performed using the suicide vector pK19mobsacB [45]. The genomic regions flanking the respective gene for homologous recombination were amplified from *C. glutamicum* ATCC13032 as described elsewhere [46] using the respective primer pairs (Table S1). Purified PCR products were assembled and simultaneously cloned into BamHI restricted pK19mobsacB by Gibson Assembly resulting in Table S1 listed plasmids. Targeted gene deletion was carried out via two-step homologous recombination as described previously [45]. Transfer of the suicide vectors was carried out via transconjugation using E. *coli* S17-1 as donor strain [40]. For the first recombination event, integration of the vector in the targeted flanking regions was selected via kanamycin resistance. Integration of the vector into the genome lead to a sucrose sensitivity due to the expression of sacB, encoding a levansucrase. During the second recombination, the suicide vector is excised, and sucrose-resistant clones could be verified by PCR by using respective combination of UF fw and DF rev primers or verification primers (Table S1).

### 2.3. Protein Analytics

For protein purification fresh *E. coli* BL21 (DE3) carrying either pET16b-*dpkA* or pET16b-*dpkA*^P262M141L^ were inoculated to an OD_600_ 0.05 in 500 mL in 2 L baffled flasks. Gene expression was induced by addition of 1 mM IPTG when OD_600_ 0.5−0.6 was reached and flasks were transferred to 20 °C and 180 rpm. After 3.5 h of expression, cells were harvested by centrifugation and the pellets were stored at −20 °C for further use. Following steps were handled on ice. The pellets were resuspended in TNGI5 buffer (pH 7.9, 20 mM Tris/HCL, 300 mM NaCl, 5 g L^−1^ glycerol and 5 mM imidazole). For protease inhibition 1 mM PMSF was added. Cell disruption was performed by sonication (UP 200S, Dr. Hielscher GmbH, Teltow, Germany) at an amplitude of 60% and a duty cycle of 0.5 s for 4 min. To obtain cell free extracts the cell suspension was centrifuged (20,200× *g*, 60−90 min, 4 °C). The proteins carrying 10xHis-tag were purified via Ni-NTA resin (Qiagen, Venlo, Netherlands) according to the manufacturer. For elution of targeted protein TNGI buffer containing 200 mM imidazole was used. Protein concentration was determined using Bradford reagent with bovine serum as a reference.

The determination of the reductive *N-*methylation activity of DpkA was carried out as described previously [28]. The consumption of NADPH was measured over time (3 min) at 340 nm in a reaction mixture of 100 mM glycine buffer (pH 10.0), 60 mM MMA, 10 mM phenylpyruvate or pyruvate and 0.3 mM NADPH in a total volume of 1 mL. The measurements were performed at least in triplicates. The Michaelis constants (K_m_) were determined using Origin with the add on “Enzymatic kinetics”. Catalytic efficiency was calculated with respect to Michealis Menten [47] (MW DpkA: 35.14 kDa). Specific activity of DpkA is shown in units (U), where one unit is defined as the amount of enzyme, required to convert 1 µmol substrate in one minute.

### 2.4. Quantification of Amino Acids and Organic Acids

Extracellular amino acids and carbohydrates were quantified by high-performance liquid chromatography (HPLC) (1200 series, Agilent Technologies Deutschland GmbH, Böblingen, Germany). The culture supernatants were collected at different time points and centrifuged (20,200× *g*, 10 min) for HPLC analysis.

Shikimate and phenylpyruvate were detected with an amino exchange column (Aminex, 300 mm × 8 mm, 10µm particle size, 25 Å pore diameter, CS Chromatographie Service GmbH) under isocratic conditions for 22 min at 37 °C with 10 mM sulfuric acid and a flow rate of 0.6 mL min^−1^. The detection of shikimate and phenylpyruvate was carried out with a Diode Array Detector (DAD, 1200 series, Agilent Technologies) at 210 nm. Glucose and xylose were detected by a refractive index detector (RID G1362A, 1200 series, Agilent Technologies).

For the detection of *N-*methylphenylalanine and *N*-methylalanine, the samples were derivatized with fluorenylmethyl chloroformate (FMOC) (Karl Roth, Karlsruhe, Germany) according to published methods [48]. Proline was used as internal standard.

The separation of the FMOC-derivatized amino acids was performed on a system consisting of a pre-column (LiChrospher 100 RP18 EC-5m (40 × 4 mm) and a main column (LiChrospher 100 RP18 EC-5m (125 × 4 mm) with an initial flow rate of 0.75 mL min^−1^ for 5 min and 1.3 mL min^−1^ subsequently. As mobile phase, the eluents sodium acetate (50 mM, pH 4.2) (A) and acetonitrile (B) were used with the following gradient: 0 min 38% B, 5 min 38% B, 7 min 41% B, 14 min 57% B, 16 min 76% B, 17 min 76% B and 19 min 38% B. The detection was carried out with a fluorescence detector (FLD) with an excitation of 250 nm and emission of 410 nm.

## 3. Results

### 3.1. Assessing the Suitability of C. glutamicum for NMePhe Production

**Figure 2:**
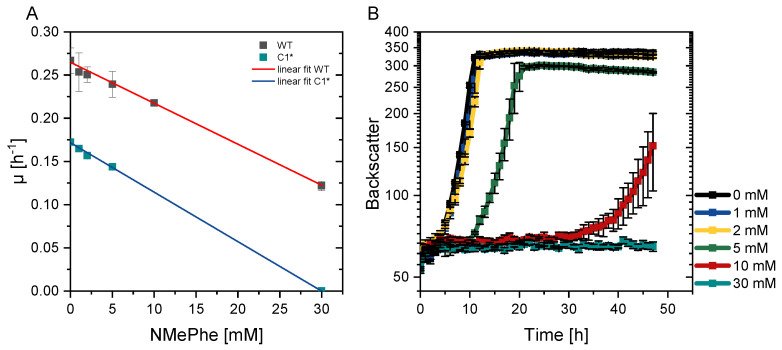
Maximal specific growth rates of *C. glutamicum* wild type (grey) and C1* (blue) in the presence of different NMePhe concentrations (**A**) and the corresponding growth curves shown for C1* (**B**). Half-maximal growth rates were obtained by extrapolation. The cultivations were performed in standard CGXII medium with 10 g L^−1^ glucose as sole carbon source in a BioLector system. Means and standard deviation are shown of technical triplicates.

A suitable production host should tolerate high product concentrations without degrading the product. Previously, it was shown that the *N*-alkyldonor MMA used here only has minor impacts on growth of *C. glutamicum* wild type [28]. First, it was tested if *C. glutamicum* wild type utilized the product of interest, NMePhe, as sole carbon or nitrogen source. Therefore, growth experiments with CGXII minimal medium containing either 30 mM ammonium sulfate and 17 mM urea or 30 mM NMePhe as sole nitrogen sources or either 30 mM glucose or 30 mM NMePhe as sole carbon sources. NMePhe neither supported growth of *C. glutamicum* as sole carbon nor as nitrogen source (data not shown). Next, to test for possible product toxicity *C. glutamicum* wild type and C1*, a genome-reduced platform strain derived from the wild type [42], were grown in the BioLector microcultivation system using glucose, minimal medium with NMePhe concentrations in the range of 0 to 30 mM. NMePhe affected the growth of *C. glutamicum* wild type and C1* similarly with 27 and 24 mM (about 4.5 g L^−1^) reducing the growth rate to half-maximal although the wild type grew faster than C1* under all conditions tested (Figure 2). Besides reducing the maximal specific growth rate, NMePhe also elongated the lag phase, which, for example, took about 8 h and 30 h in the presence of 5 and 10 mM NMePhe, respectively **(**Figure 2B). Taken together, *C. glutamicum* appears to be suitable for growth-coupled production of NMePhe to titers in the g L^−1^ range.

### 3.2. Exchanging Two Amino Acid Residues of DpkA to Affect Preference of the Substrates Pyruvate and Phenylpyruvate

**Figure 3:**
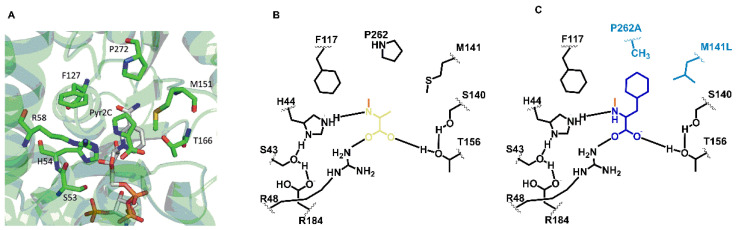
Schematic representations of the substrate binding site of DpkA or DpkA^P262A,M141L^ with different 2-oxoacids as substrates. (**A**) Active site of DpkA from *Pseudomonas syringae* (PDB: 2CWH). The native substrate pyrroline-2-carboxylate (Pyr2C; carbon atoms in green) and the cofactor NADPH (carbon atoms in light gray) are bound to the active site. The pyrrole ring of Pyr2C is recognized by the three amino acid residues Phe117, Pro262, and Met141 (carbon atoms in green). (**B**) Schematic view of the active site of DpkA with the 2-oxoacid substrate pyruvate (brown). (**C**) Schematic view of the active site of DpkA^P262A,M141L^ with the 2-oxoacid substrate phenylpyruvate (blue) (Adapted from [29]). All substrates are in their *N*-methylaminated forms.

Besides its native substrate Δ-1-piperideine-2-carboxylate, DpkA accepts pyruvate as 2-oxo acid substrate for methylamination, which has been used for enabling a pyruvate producing *C. glutamicum* strain to efficiently produce *N*-methyl- l-alanine by heterologous expression of *dpkA* [28]. Introduction of an amino acid exchange in the enzyme’s substrate binding pocket (DpkA^F117L^) proved valuable to produce sarcosine and *N*-ethyl-glycine [29]. Under the assumption that the intracellular concentration of the central intermediate pyruvate would be higher than that of the ultimate l-phenylalanine precursor molecule phenylpyruvate, it was tested here if the introduction of two amino changes changed the relative activity with phenylpyruvate and pyruvate. The two 2-oxoacid substrates differ by the size of their substituents: a small methyl group in pyruvate as compared to the large phenyl group of phenylpyruvate. To ease accommodation of the larger phenyl substituent in the substrate binding pocket, the prolyl residue 262 and the methioninyl residue 141 were replaced by alanyl and leucyl residues, respectively, in DpkA ^P262A,M141L^ (Figure 3).

DpkA and DpkA^P262A,M141L^ were produced as His-tagged proteins, purified and their kinetic parameters towards pyruvate and phenylpyruvate were determined as described in Materials and Methods (Table 2; Supplementary Materials Figure S2). Wild-type DpkA showed a clear preference for the pyruvate as substrate as the specific activity with pyruvate (32.7 ± 5 U mg^−1^) was about thirteen times higher than with phenylpyruvate (2.5 ± 0.2 U mg^−1^). Previously, DpkA was described to accept pyruvate 6 to 7 times better than phenylpyruvate [49], a discrepancy that may be due to different assay conditions (sulfate vs. glycine buffer, different NADPH concentrations). Determination of the K_m_ for phenylpyruvate revealed that it was lower than for pyruvate (Table 2), whereas the catalytic efficiency was 2.5 higher for pyruvate than for phenylpyruvate (Table 2). The amino acid exchanges present in DpkA^P262A,M141L^ reduced the catalytic efficiency for both substrates. The specific activity with pyruvate was reduced about three-fold, the K_m_ was increased about two-fold and the catalytic efficiency with pyruvate was reduced seven-fold (Table 2). With an almost unchanged specific activity with phenylpyruvate, a two-fold increased K_m_ for this substrate was detected. DpkA^P262A,M141L^ showed a catalytic efficiency for phenylpyruvate that was as high as that for pyruvate (Table 2).

It has to be noted that the amino acid exchanges present in DpkA^P262A,M141L^ did not improve, but maintained the catalytic efficiency with phenylpyruvate, and importantly reduced that with pyruvate. Thus, even in the presence of non-negligible concentrations of pyruvate in the *C. glutamicum* cell, DpkA^P262A,M141L^ may prove useful for *N*-alkylamination of phenylpyruvate to yield NMePhe.

### 3.3. Metabolic Engineering of C. glutamicum for Efficient Provision of Phenylpyruvate as Precursor

**Figure 4:**
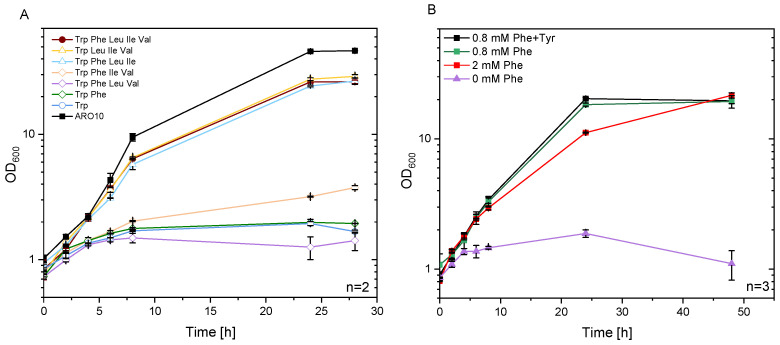
Growth of *C. glutamicum* strain ARO11 and ARO12 in standard minimal medium with various combination of amino acids. CGXII minimal medium, with 40 g L^−1^ glucose as sole carbon source, was supplemented with tryptophan as single amino acid (open circle, blue), or with the addition of l-phenylalanine (open diamond, green), or l-phenylalanine, l-leucine, and l-valine (open diamond, purple), or l-phenylalanine, l-isoleucine and l-valine (open diamond, yellow), or l-phenylalanine, l-leucine and l-isoleucine (open triangle, blue). Furthermore, the addition of l-leucine, l-isoleucine and l-valine was tested (open tringle, yellow) and the supplementation of all five amino acids (closed circle, red). ARO10 was used as a control strain. All means are shown of technical duplicates (**A**). Growth behavior of ARO12 in minimal medium (containing l-tryptophan, l-leucine, and l-isoleucine) supplemented with 0 mM (triangle, purple), 0.8 mM (square, green) 2 mM (closed square, red) phenylalanine, and 0.8 mM phenylalanine and tyrosine (square, black) is depicted in (**B**). Means and standard deviations are given of technical triplicates.

Efficient provision of phenylpyruvate was expected to result from a high flux into the shikimate pathway coupled with conversion of chorismate solely to phenylpyruvate, but not to l-tryptophan, l-tyrosine or l-phenylalanine. As a base strain, *C. glutamicum* ARO9 was used, which was constructed for overproduction of anthranilate, an intermediate of the shikimate pathway, and *N*-methylated anthranilate [36]. To abolish synthesis of l-tryptophan, the anthranilate synthase genes *trpEG* were deleted. The resulting strain ARO10 was auxotrophic for l-tryptophan (Figure S1). Biosynthesis of l-phenylalanine involves transamination of phenylpyruvate by phenylalanine aminotransferase AroT and branched-chain amino acid aminotransferase IlvE. To abolish their biosynthesis and to accumulate phenylpyruvate, the transaminase genes were deleted (Δ*ilvE* in ARO11 and Δ*ilvE* Δ*aroT* in ARO12). Strain ARO11 was auxotrophic for the branched-chain amino acids l-leucine and l-isoleucine as well as for l-tryptophan (Figure 4A). In addition, strain ARO12 was auxotrophic for l-phenylalanine (Figure 4B).

To reduce biosynthesis of pyruvate, which competes with phenylpyruvate for DpkA, the pyruvate kinase gene *pyk* was deleted in strain ARO12. The resulting strain ARO13 could grow with the PTS sugar glucose, but was not able to grow with non-PTS sugars such as maltose (data not shown), since only the PTS converts PEP to pyruvate in the absence of pyruvate kinase.

To improve conversion of shikimate to phenylpyruvate, two plasmids were constructed. Conversion of chorismate to phenylpyruvate was targeted by a feed-back resistant variant of the bifunctional chorismate mutase/prephenate dehydratase from *E. coli*. Its gene, *pheA*^FBR^, was cloned into the vector pEKEx3 and used to transform *C. glutamicum* strains ARO11, ARO12 and ARO13 to yield strain ARO11A, ARO12A and ARO13A, respectively (Table 1). In addition, conversion of shikimate to shikimate-3-phosphate was targeted by shikimate kinase from *Methanococcus jannaschii.* Its gene, *aroK*, was expressed as synthetic operon with *pheA*^FBR^ in plasmid pEKEx3-*pheA*^FBR^-*aroK*_MJ_. Transformants of ARO11, ARO12 and ARO13 with pEKEx3-*pheA*^FBR^-*aroK*_MJ_ were named ARO11B, ARO12B and ARO13B, respectively. Due to expression of *aroK*_MJ_, the strains ARO11B, ARO12B and ARO13B accumulated less shikimate than strains ARO11A, ARO12A and ARO13A (Figure S3). Notably, shikimate accumulation was reduced about four-fold and about fourteen-fold comparing strains and ARO12A (0.2 ± 0.01 g L^−1^) with ARO12B (0.05 ± 0.01 g L^−1^) and ARO13A (1.4 ± 0.01 g L^−1^) with ARO13B (0.1 ± 0.01 g L^−1^), respectively (Figure S3).

### 3.4. DpkA Mediated Methylamination of Phenylpyruvate Yielded NMePhe

**Figure 5:**
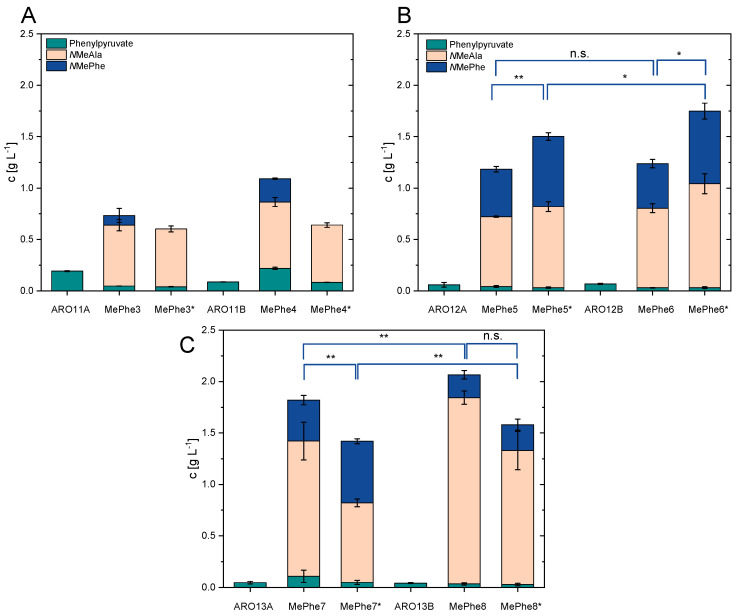
Production of phenylpyruvate (cyan), NMeAla (orange) and NMePhe (blue) by various *C. glutamicum* strains. Production by strains derived from ARO11 is depicted in (**A**), from ARO12 in (**B**) and from ARO13 in (**C**) after cultivationin Duetz-plates in CGXII minimal medium containing 50% nitrogen source and 20 g L^−1^ glucose as sole carbon source for 72 h. As methyl-donor 0.35 M MMA was applied. Means and standard deviations are depicted from technical triplicate cultures. Significance has been determined for NMePhe concentrations based on a two-sided unpaired Welch-t test (*: *p* ≤ 0.05, **: *p* ≤ 0.01, n.s.: not significant).

To enable NMePhe production plasmid-borne expression of *dpkA* was used. A suite of strains was transformed using either pVWEx1-*dpkA*_RBS^opt^ or pVWEx1-*dpkA*^P262M141L^ to compare native DpkA with the variant DpkA^P262M141L^. Transformants of ARO11A with pVWEx1-*dpkA*_RBS^opt^ and pVWEx1-*dpkA*^P262M141L^ were named NMePhe3 and NMePhe3*, respectively. Transformants of strains ARO11B, ARO12A, ARO12B, ARO113A and ARO13B were named accordingly (Table 1). To test for NMePhe production, these strains were cultivated in Duetz-plates in CGXII minimal medium with 20 g L^−1^ glucose as sole carbon source, a nitrogen concentration reduced to 50% and 0.35 M MMA (Figure 5).

A total of five conclusions could be drawn from the metabolic engineering approaches and strain NMePhe5* was selected as the most promising strain for NMePhe production. First, only strains expressing *dpkA* or *dpkA*^P262M141L^ produced NMeAla and NMePhe and they accumulated less phenylpyruvate than the precursor strain without DpkA (Figure 5). Second, NMePhe5, NMePhe5*, NMePhe6, and NMePhe6* that carry both transaminase gene deletions (Δ*ilvE* Δ*aroT*) produced more NMePhe than their isogenic precursors that only carry the deletion Δ*ilvE* (compare Figure 4B with Figure 4A). Third, the Δ*pyk* deletion did not increase NMePhe production, but unexpectedly led to more NMeAla production (compare Figure 5C with Figure 5B). Fourth, when comparing, e.g., strain NMePhe6 that expresses the synthetic *pheA*^FBR^-*aroK*_MJ_ operon with its isogenic precursor NMePhe5 that only expresses *pheA*^FBR^ it became evident that the additional expression of *aroK*_MJ_ increased NMeAla titers, but not NMePhe production. Fifth, the comparison of, e.g., strains NMePhe5* with NMePhe5 as well as NMePhe6* with NMePhe6 (Figure 5B) revealed that DpkA^P262M141L^ was suited better for NMePhe production than native DpkA as strains NMePhe5* and NMePhe6* (with DpkA^P262M141L^) produced more NMePhe than strains NMePhe5 and NMePhe6 (with native DpkA). Of all the strains tested, strain NMePhe5* showed the highest NMePhe production (0.68 ± 0.03 g L^−1^). Thus, although metabolic engineering of precursor supply and use of variant DpkA^P262M141L^ was successful to enable NMePhe production, NMeAla (0.78 ± 0.05 g L^−1^) remained a significant by-product.

### 3.5. Improvement of NMePhe Production and Reduction of NMeAla

**Figure 6:**
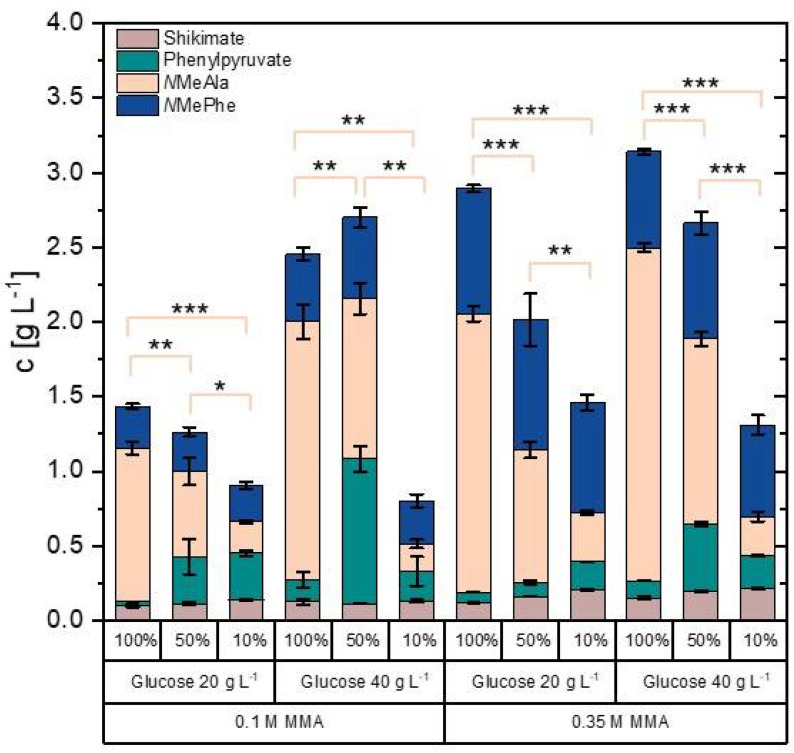
Production of NMePhe (blue), NMeAla (orange), phenylpyruvate (cyan), and shikimate (light brown) by *C. glutamicum* strain NMePhe5* with different culture media compositions. Strain NMePhe5* was grown using CGXII media with the indicated concentrations of alkylamine donor (0.1 M and 0.35 M MMA), carbon source (20 g L^−1^ and 40 g L^−1^ glucose), and nitrogen source (10%, 50% and 100% of the concentrations of the nitrogen sources urea and ammonium sulfate). Means and standard deviations of triplicate cultures are depicted. Significance of reduction of the by-product NMeAla (orange) has been determined based on a two-sided unpaired Welch t-test (*: *p* ≤ 0.05, **: *p* ≤ 0.01, ***: *p* ≤ 0.001). More detailed statistical analysis can be found in Supplementary Figure S5.

Media composition was varied in order to assess the effect on NMePhe production and on the product to by-product ratio. To this end, the culture media concentrations of MMA as alkylamine donor (0.1 M and 0.35 M), the carbon source (20 g L^−1^ and 40 g L^−1^ glucose), and the nitrogen source (10%, 50% and 100% of the concentrations of the nitrogen sources urea and ammonium sulfate) were varied and growth (Figure S4) and production of NMeAla, shikimate, phenylpyruvate, and NMePhe were monitored using strain NMePhe5* (Figure 6).

Regarding the alkylamine donor MMA, NMePhe production was higher with 0.35 M MMA than with 0.1 M MMA (Figure 5). With 0.35 M MMA, NMePhe production did not increase when the carbon source concentration was increased from 20 g L^−1^ to 40 g L^−1^ glucose (Figure 6). The CGXII minimal medium contains a very high concentration of the nitrogen sources urea and ammonium sulfate as it was optimized for production of lysine, a product that contains two nitrogen atoms [44]. The nitrogen atom in NMePhe derives from the alkylamine donor MMA, whereas the precursor phenylpyruvate that is synthesized de novo by *C. glutamicum* does not contain a nitrogen atom. Thus, the nitrogen source is only required for biomass formation. With 0.35 M MMA and 20 g L^−1^ glucose, NMePhe production was comparable regardless whether the concentration of the nitrogen source was set to 10%, 50% or 100% (Figure 6). However, the formation of NMeAla as by-product was positively correlated with the nitrogen source concentration: from 1.9 ± 0.5 g L^−1^ at 100% to 0.33 ± 0.02 g L^−1^ at 10% (Figure 6). Taken together, adaptation of the medium reduced by-product formation considerably. With 0.35 M MMA, 20 g L^−1^ glucose, and 10% nitrogen content of the culture medium, NMePhe was produced to a titer of 0.73 ± 0.05 g L^−1^ with a volumetric productivity of 0.01 g L^−1^ h^−1^ at a yield of 0.052 g g^−1^ glucose (Figure 6).

### 3.6. Establishing NMePhe Production from the Alternative Feedstock Xylose

**Figure 7:**
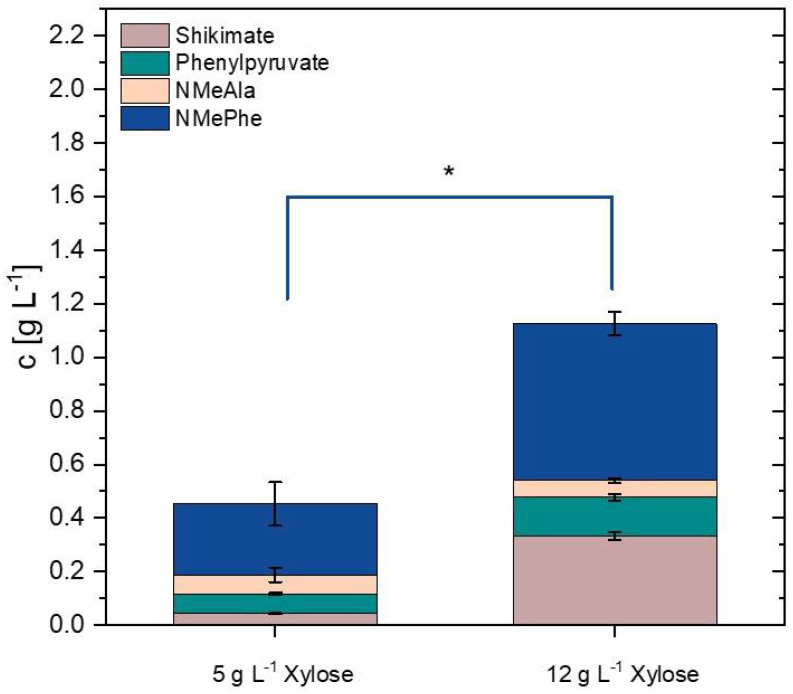
Xylose-based production of NMePhe (blue), NMeAla (orange) phenylpyruvate (cyan), and shikimate (light brown) by *C. glutamicum* strain MePhe9*. After growth in Duetz-plates for 72 h using CGXII medium containing 50% nitrogen, 0.35 M MMA and either 5 or 12 g L^−1^ xylose as sole carbon source, the concentrations of NMePhe, NMeAla, phenylpyruvate and shikimate were determined in culture supernatants. Means and standard deviations from triplicate cultures are depicted. Significance has been determined for NMePhe concentrations based on a two-sided unpaired Welch-t test (* *p* ≤ 0.05).

There is an increasing demand for carbon sources for biotechnological processes that do not compete with use as food or feed. Second generation feedstocks such as lignocellulosic hydrolysates contain xylose besides glucose. We have previously established that heterologous expression of the xylose isomerase gene *xylA* from *Xanthomonas campestris* and overexpression of the endogenous xylulokinase gene *xylB* enables efficient utilization of xylose as carbon source for growth and production [50,51]. Therefore, strain NMePhe5* was transformed with plasmid pECXT-*Psyn*-*xylAB* to yield strain MePhe9*. With 0.35 M MMA and 10% nitrogen content in CGXII minimal medium, xylose-based production of NMePhe was tested with either 5 or 12 g L^−1^ xylose as sole carbon source. With 5 g L^−1^ xylose, strain MePhe9* produced 0.3 ± 0.1 g L^−1^ NMePhe and accumulated 0.07 ± 0.03 g L^−1^ NMeAla and 0.07 ± 0.01 g L^−1^ phenylpyruvate as by-products (Figure 7). With 12 g L^−1^ xylose, shikimate was the major by-product (0.3 ± 0.01 g L^−1^), while accumulation of NMeAla remained low (0.06 ± 0.01 g L^−1^). Under this condition, 0.6 ± 0.04 g L^−1^ NMePhe were produced with a volumetric productivity of 0.008 g L^−1^ h^−1^ and a yield of 0.05 g g^−1^ xylose (Figure 7). Thus, xylose-based as well as and glucose-based production of NMePhe proceeded with very comparable product yields on substrate.

## 4. Discussion

In this study, we achieved sustainable fermentative production of NMePhe de novo from glucose and xylose as carbon sources. The process was based on reductive methylamination of phenylpyruvate. *C. glutamicum* was engineered for provision of phenylpyruvate, which was converted with MMA to NMePhe using *P. putida* Δ-1-piperideine-2-carboxylate reductase DpkA. The variant DpkA^P262A,M141L^ proved beneficial because less NMeAla was synthesized as by-product from pyruvate.

The proof-of-principle of the de novo fermentative process described here differs from enzyme biocatalysis or whole-cell biotransformation as it does not require the addition of costly phenylpyruvate as substrate, but rather operates with a sugar as carbon source and *N*-alkyldonor MMA added to a mineral salts medium. Previously, an enzyme cascade with *P. putida* DpkA and *B. subtilis* glucose dehydrogenase was used to convert phenylpyruvate and MMA to 16 g L^−1^ NMePhe with excellent yield (98%) and ee (>99%) [52]. NADPH added in sub-stoichiometric amounts had to be regenerated by oxidation of glucose to gluconolactone by glucose dehydrogenase. Upon repeated addition of the substrates in a scale-up experiment the yield was dramatically reduced, which was attributed to inactivation of glucose dehydrogenase [52]. By contrast, although not studied here, the scale-up of fermentative *C. glutamicum* processes typically benefits from the experience of 60 years of amino acid production at the million-ton scale using this bacterium [53,54].

DpkA shows a relatively broad 2-oxoacid substrate spectrum [49]. Previously, we have shown that *C. glutamicum* strains engineered to accumulate the 2-oxoacid substrate of choice for the DpkA dependent alkylamination are suited to produce one major *N*-alkylated amino acid [22,23,24,25,26,27,28,29]. Unlike biocatalysis approaches where only a single 2-oxoacid is provided as substrate, *C. glutamicum* metabolism comprises a number of 2-oxoacids as metabolites, e.g., pyruvate, oxaloacetate or 2-oxoglutarate. While the latter 2-oxoacids are not substrates for DpkA, pyruvate is accepted well [49], which was employed for NMeAla production [28]. Production of NMeAla as by-product disturbed production of *N*-methylated or *N*-ethylated glycine derivates, which was achieved after having shown that DpkA accepts glyoxylate as substrate [27,29]. Similarly, conversion of the major intracellular metabolite pyruvate in *C. glutamicum* cells to NMeAla occurred during NMePhe production as described here. This limitation could be partly overcome by medium optimization (Figure 6 and [27,28]).

Changing the substrate specificity of DpkA such that only the 2-oxoacid of interest is converted would overcome the problem due to multiple 2-oxoacids present as metabolites in the *C. glutamicum* cell. However, this has not yet been achieved. The variant constructed here, DpkA^P262A,M141L^, was characterized by reduced catalytic efficiency with pyruvate, but the catalytic activity with phenylpyruvate was not increased (Table 2). While the tendency is correct, a preference of phenylpyruvate over pyruvate was, however, not observed. A similar result was obtained earlier with variant DpkA^F117L^, which exhibited higher catalytic efficiency with glyoxylate and monoethylamine than native DpkA; however, the catalytic efficiency with pyruvate and MMA was also increased. Thus, further research activities have to be dedicated to change the substrate specificity of DpkA. These may include random, rational or semi-rational approaches to enzyme engineering [55,56,57]. Taking the structural information of an IRED into account to generate mutant libraries allowed to change the cofactor specificity of that enzyme from NADPH to NADH [58]. DpkA also accepts the C2 compound monoethylamine for *N*-alkylamination of the short 2-oxoacids glyoxylate and pyruvate [27,29,59]. However, the steric requirement of the phenyl substituent in the substrate binding pocket of DpkA provides little space to accommodate monoethylamine instead of MMA. Thus, *N*-alkylamination of phenylpyruvate with an alkylamine other than MMA most likely requires more profound rearrangement of the catalytic center and/or substrate binding pocket of DpkA.

The metabolic engineering strategy followed here to provide the precursor phenylpyruvate largely relies on optimizing entry into and conversion in the shikimate pathway. This approach enabled production of aromatic compounds to about g L^−1^ titers: 1.4 g L^−1^ shikimate, 3.0 g L^−1^ anthranilate, 0.5 g L^−1^ N-methyl-anthranilate [36] and 0.73 ± 0.05 g L^−1^ NMePhe in this study. Notably, the yield of NMePhe on glucose of 0.052 g g^−1^ was one order of magnitude higher than that for *N*-methylantranilate (4.8 mg g^−1^ glucose) [36]. However, recent examples of metabolic engineering of *C. glutamicum* to produce l-tryptophan [60], pHBA [61,62], *cis*-muconic acid [63], phenylpropanoids [64], and protocatechuic acid (PCA) [65] led to much higher product titers and yields. Notably, PCA production amounted to a titer of 82.7 g L^−1^ with a yield of 32.8% (mol mol^−1^) from glucose in growth-arrested cell reaction [65]. Thus, further metabolic engineering and process optimization including the use of non-growing cells in a whole-cell biotransformation as realized for PCA [65] is an encouraging option to boost NMePhe production in the future.

The strains constructed here also allowed for xylose-based production of NMePhe. Xylose is a major constituent of lignocellulose hydrolysates [66] and is an abundant and renewable feedstock for many biotechnological processes [67]. Xylose cannot be catabolized by *Saccharomyces cerevisiae* [66,68], *Zymomonas mobilis* [69] or *C. glutamicum* [50,51]. However, *C. glutamicum* was engineered for xylose utilization via the Weimberg pathway [70], in which xylose is exclusively oxidized to 2-oxoglutarate, a major intermediate of the TCA cycle, without carbon loss [71]. This approach is suitable for xylose-based production of chemicals that derive from 2-oxoglutarate. Production of NMePhe, however, requires PEP and the pentose phosphate pathway intermediate E4P as precursors; thus, a xylose catabolic pathway entering the PPP is beneficial. In this respect, the so-called isomerase pathway for xylose utilization is conducive. This pathway has been realized in *C. glutamicum* and applied to xylose-based production [50,51,71,72]. Xylose-based NMePhe production was observed upon plasmid-borne expression of the xylose isomerase gene *xylA* from *Xanthomonas campestris* and the endogenous xylulokinase gene *xylB* (Table 1). Notably, the NMePhe yield on glucose and on xylose were comparable (about 0.05 g g^−1^). The low volumetric productivity of 0.008 g L^−1^ h^−1^ may possibly be increased by transport engineering [73]. Xylose is taken up by an unidentified transporter in *C. glutamicum;* however, derepression of the *myo*-inositol permease gene *iolT1*, which also accepts fructose, glucose and xylose [74]*,* may accelerate xylose-based NMePhe production. Alternatively, heterologous expression of *araE*, encoding the uptake system for arabinose and xylose uptake in *C. glutamicum* ATCC 31831 [75], may be used. Fermentative production of NMePhe from arabinose, the second most abundant pentose sugar present in lignocellulosic hydrolysates, may be realized by heterologous expression of *araBAD* from *E. coli* [75,76]. Access to glycerol [77,78], a by-product of the biodiesel process, amino sugars such as glucosamine [79], *N*-acetyl-glucosamine [80] or *N*-acetyl-muraminate [81] that derive, e.g., from bacterial and fungal cell walls is not important here since entry of glycerol into the PPP is energy requiring and since the nitrogen of NMePhe derives from MMA; thus, amino sugars do not provide an advantage over nitrogen-free feedstocks.

Taken together, this study describes the first proof-of-principle of de novo production of NMePhe from glucose or xylose and MMA. To achieve an economically viable process, further optimization of titers, yields and volumetric productivities is required.

## Figures and Tables

**Table 1:** Used strains in this work.

Strain	Description	Source
*Escherichia coli*		
S17-1	*recA pro hsdR* RP4-2-Tc::Mu-Km::Tn7	[40]
DH5α	*F-thi-1 endA1 hsdr17(r-, m-) supE44 1lacU169 (* *Φ80lacZ1M15) recA1 gyrA96*	[41]
BL21(DE3)	*fhuA2 [lon] ompT gal (λDE3) [dcm]* Δ*hsdS λ DE3* = *λ sBamHIo* Δ*EcoRI-B int::(lacI::PlacUV5::T7°gene1) i21* Δ*nin5*	Novagen
*Corynebacterium glutamicum*		
WT	*C. glutamicum* wild type, ATCC 13032	ATCC
C1*	Genome-reduced strain derived from *C. glutamicum* WT	[42]
ARO9	Δ*vdh*::*P_ilvC_-aroG*^D146N^ Δ*ldhA* Δ*aroR*::*P_ilvC_-aroF* Δ*qsuBCD*::*P_tuf_-qsuC* Δ*ppc*::*P_sod_-aroB* Δ*P_tkt_*::*P_tuf_-tkt* Δ*iolR*::*P_tuf_-aroE*	[36]
ARO10	Δ*trpEG* mutant of ARO9	This work
ARO10A	ARO10 carrying pEKEx3-*pheA*^FBR^ and pVWEx1	This work
ARO10B	ARO10 carrying pEKEx3-*pheA*^FBR^-*aroK* and pVWEx1	This work
ARO11	Δ*ilvE* mutant of ARO10	This work
ARO11A	ARO11 carrying pEKEx3-*pheA*^FBR^ and pVWEx1	This work
ARO11B	ARO11 carrying pEKEx3-*pheA*^FBR^-*aroK* and pVWEx1	This work
ARO12	Δ*aroT* mutant of ARO11	This work
ARO12A	ARO12 carrying pEKEx3-*pheA*^FBR^ and pVWEx1	This work
ARO12B	ARO12 carrying pEKEx3-*pheA*^FBR^-*aroK* and pVWEx1	This work
ARO13	Δ*pyk* mutant of ARO12	This work
ARO13A	ARO13 carrying pEKEx3-*pheA*^FBR^ and pVWEx1	This work
ARO13B	ARO13 carrying pEKEx3-*pheA*^FBR^-*aroK* and pVWEx1	This work
MePhe1	ARO10 carrying pEKEx3-*pheA*^FBR^ and pVWEx1-*dpkA*-RBS^opt^	This work
MePhe2	ARO10 carrying pEKEx3-*pheA*^FBR^-*aroK* and pVWEx1-*dpkA*-RBS^opt^	This work
MePhe3	ARO11 carrying pEKEx3-*pheA*^FBR^ and pVWEx1-*dpkA*-RBS^opt^	This work
MePhe3*	ARO11 carrying pEKEx3-*pheA*^FBR^and pVWEx1- *dpkA*^P262M141L^	This work
MePhe4	ARO11 carrying pEKEx3-*pheA*^FBR^-*aroK* and pVWEx1-*dpkA*-RBS^opt^	This work
MePhe4*	ARO11 carrying pEKEx3-*pheA*^FBR^-*aroK* and pVWEx1-*dpkA*^P262M141L^	This work
MePhe5	ARO12 carrying pEKEx3-*pheA*^FBR^ and pVWEx1-*dpkA*-RBS^opt^	This work
MePhe5*	ARO12 carrying pEKEx3-*pheA*^FBR^and pVWEx1-*dpkA*^P262M141L^	This work
MePhe6	ARO12 carrying pEKEx3-*pheA*^FBR^-*aroK* and pVWEx1-*dpkA*-RBS^opt^	This work
MePhe6*	ARO12 carrying pEKEx3-*pheA*^FBR^-*aroK* and pVWEx1-*dpkA*^P262M141L^	This work
MePhe7	ARO13 carrying pEKEx3-*pheA*^FBR^ and pVWEx1-*dpkA*-RBS^opt^	This work
MePhe7*	ARO13 carrying pEKEx3-*pheA*^FBR^and pVWEx1-*dpkA*^P262M141L^	This work
MePhe8	ARO13 carrying pEKEx3-*pheA*^FBR^-*aroK* and pVWEx1-*DpkA*-RBS^opt^	This work
MePhe8*	ARO13 carrying pEKEx3-*pheA*^FBR^-*aroK* and pVWEx1-*dpkA*^P262M141L^	This work
MePhe9*	MePhe5* carrying pEC-XT99A_prytt_-*xylA_Xc_-XylB_Cg_*	This work

**Table 2:** Comparison of the 2-oxoacids pyruvate and phenylpyruvate as substrates for purified 10xHis-DpkA. For specific activity measurements the reaction was assayed in a total volume of 1 mL containing 100 mM glycine buffer pH 10, 60 mM MMA, 10 mM of the respective 2-oxoacid and 0.3 mM NADPH. The consumption of NADPH was followed at 340 nm at 30 °C for 3 min. Means and standard deviations of triplicate measurements are given.

Enzyme	2-Oxoacid Substrate	K_m_ (mM)	Specific Activity (U mg^−1^)	k_ca__t_ (s^−1^)	CatalyticEfficiency (s^−1^ mM^−1^)
DpkA	Pyruvate	5 ± 1	32.7 ± 5	19.2	3.8
DpkA	Phenylpyruvate	1 ± 0.3	2.5 ± 0.2	1.5	1.5
DpkA^P262A,M141L^	Pyruvate	11.4 ± 1	9.5 ± 0.5	5.6	0.5
DpkA^P262A,M141L^	Phenylpyruvate	2 ± 0.3	2 ± 0.2	1.2	0.6

## Data Availability

All data are present in the manuscript and its supplement.

## Supplementary Materials

The following are available online at https://www.mdpi.com/article/10.3390/microorganisms9040824/s1, Table S1: Used plasmids and oligonucleotides in this work, Figure S1: Verification of tryptophan auxotrophy of ARO10, Figure S2: Michaelis-Menten kinetics for DpkA and DpkA^P262A,M141L^ with pyruvate and phenylpyruvate, Figure S3: Production of shikimate by *C. glutamicum* ARO and MePhe strains, and Figure S4: Growth of *C. glutamicum* strain NMePhe5* with different culture media compositions, Figure S5: Production of NMePhe (blue), NMeAla (orange), phenylpyruvate (cyan), and shikimate (light brown) by C. glutamicum strain NMePhe5* with different culture media compositions.
