# Supplementary material for: Sustainable Production of N-methylphenylalanine by Reductive Methylamination of Phenylpyruvate Using Engineered Corynebacterium glutamicum
Source: Microorganisms. 2021 Apr 13;9(4):824. doi: 10.3390/microorganisms9040824 (PMC8070496; doi:10.3390/microorganisms9040824)
Supplement: Supplementary file 1 [file microorganisms-09-00824-s001.pdf]

Supplementary material to

Sustainable production of *N*-methylphenylalanine by reductive methylamination of phenylpyruvate using engineered *Corynebacterium glutamicum*

Anastasia Kerbs[1], Melanie Mindt[2], Lynn Schwardmann[1] and Volker F. Wendisch [1,*]

[1] Genetics of Prokaryotes, Faculty of Biology & CeBiTec, Bielefeld University, Germany; anastasia.kerbs@uni-bielefeld.de (A.K.), l.schwardmann@uni-bielefeld.de (L.S.)
[2] BU Bioscience, Wagenigen University & Research, 6700AA Wageningen, The Netherlands; melanie.mindt@wur.nl (M.M.)
[*] Correspondence: Volker.wendisch@uni-bielefeld.de (V.W.); Tel.: +49-521-106-5611

Table S1 : Used plasmids and oligonucleotides in this work

Plasmids and Oligonucleotides	Relevant characteristics	Source
**Plasmids**		
pET-16b	Amp^R, production of *N*-terminal 10xHis-tagged proteins in *E. coli* (pBR322 oriV*E.c.*, PT7, *lacI*)	Novagen
pET-16b-*dpkA*^{P262A,M141L}	Amp^R, pET-16b expressing *dpkA* from P. *putida* KT2440 with amino acid exchange from proline to alanine at position 262 and methionine to leucine at position 141 for protein purification	[1]
pK19*mobsacB*	Km^R; *E. coli*/*C. glutamicum* shuttle vector for construction of insertion and deletion mutants in *C. glutamicum* (pK18 *oriV*$_{Ec}$ *sacB lacZα*)	[2]
pK19*mobsacB-ΔtrpEG*	pK19*mobsacB* with a construct for deletion of *trpEG* (*cg3359*, *cg3360*)	This work
pK19*mobsacB-ΔilvE*	pK19*mobsacB* with a construct for deletion of *ilvE* (*cg2418*)	This work
pK19*mobsacB-ΔaroT*	pK19*mobsacB* with a construct for deletion of *aroT* (*cg0267*)	This work
pK19*mobsacB-ΔpyK*	pK19*mobsacB* with a construct for deletion of *pyK* (*cg2291*)	[3]
pK19*mobsacB*-ΔNcgl2922::P_{tuf}-*aroK*^{mj}	pK19*mobsacB* with a construct for deletion of N*cgl2922* and insertion of *aroK* from *Methanococcus jannaschii*	[4]
pEKEx3	Spec^R, P_{tac}*lacI*^{q}, pBL1*oriV*$_{Cg}$, *C. glutamicum*/*E. coli* expression shuttle vector	[5]
pEKEx3-*pheA*	Spec^R, pEKEx3 overexpressing *pheA*^{FBR} from *E. coli* K12	This work
pEKEx3-*pheA*^{FBR}	Spec^R, pEKEx3 overexpressing *pheA*^{FBR} from *E. coli* K12	This work
pEKEx3-*pheA*^{FBR}-*aroK*$_{MJ}$	Spec^R, pEKEx3 overexpressing *pheA*^{FBR} from *E. coli* K12 and *aroK* from *Methanococcus jannaschii*	This work
pVWEx1	Kan^R, P_{tac}*lacI*^{q} pHM1519 *oriV*$_{Cg}$ *C. glutamicum*/*E. coli* expression shuttle vector	[6]
pVWEx1-*dpkA*_RBS^{opt}	Kan^R, pVWEx1 overexpressing *dpkA* from *P. putida* KT2440 with start codon GTG instead of ATG and with an optimized RBS	[7]

pVWEx1-*dpkA*^{P262AM141L}	Kan^{R}, pVWEx1 overexpressing *dpkA* from *P. putida* KT2440 with amino acid exchange from proline to alanine at position 262 and methionine to leucine at position 141	[1]
pECXT-*Psyn-xylAB*	Tet^{R}, pECXT99A derivative for constitutive expression of *xylA* from *Xanthomonas campestris* and *xylB* from *C. glutamicum* from synthetic P*syn* promoter	[8]

**Oligonucleotides**	**Sequence (5′-3′)**	**Function**
trpEG UF fw	CAGGTCGACTCTAGAGGATCCGCATACTGTTGCGATGGTTG	Amplification upstream of *trpEG*
trpEG UF rv	TTTTATTAGTTCGCGAGAAGGGGATTCGTGCTCATGGGGC	Amplification upstream of *trpEG*
trpEG DF fw	GCCCCATGAGCACGAATCCCCTTCTCGCGAACTAATAAAAAAAGG	Amplification downstream of *trpEG*
trpEG DF rev	GAGCTCGGTACCCGGGGATCCTGCACATGCGCAATCGCAG	Amplification downstream of *trpEG*
trpEG g. fw	GCTGTCGGGAGTTTCCTTTG	Amplification of *trpEG*
trpEG g. rv	GGGACAGCAATGGTCCAAG	Amplification of *trpEG*
ilvE UF fw	CCTGCAGGTCGACTCTAGAGGATCCGTCGTCAAGCAAATCAGC	Amplification upstream of *ilvE*
ilvE UF rv	GGTTGATTTAGCCAACCAGTGGACCTGACAGATACACTAGTC	Amplification upstream of *ilvE*
ilvE DF fw	GACTAGTGTATCTGTCAGGTCCACTGGTTGGCTAAATCAACC	Amplification downstream of *ilvE*
ilvE DF rev	GAGCTCGGTACCCGGGGATCCTTTGGTGACGCGCAAAGTG	Amplification downstream of *ilvE*
ilvE g.fw	CGAGCGAGCAGGACAGATTC	Amplification of *ilvE*
ilvE g. rv	GAATTCTTCCGTGGCAACTC	Amplification of *ilvE*
aroT UF fw	CCTGCAGGTCGACTCTAGAGGATCCCTTAGCAAGACCGGGTGAC	Amplification upstream of *aroT*
aroT UF rv	CCAAAGACTACCCAGCATTGATATCTGCTCTAATCATGATTTACAC	Amplification upstream of *aroT*
aroT DF fw	GTAAATCATGATTAGAGCAGATATCAATGCTGGGTAGTCTTTGGCG	Amplification downstream of *aroT*
aroT DF rev	GATCCCCGGGTACCGAGCTCGGACGGTCAATGACACATCGTTC	Amplification downstream of *aroT*
aroT g.fw	AGAAGCCGGCATACCCGAAG	Amplification of *aroT*

aroT g.rv	TTGAGCTTGAGCGGAAATGC	Amplification of *aroT*
pyk ver fw	TCTTCGCTTTGTTGATGTGGGCTGAC	Verification of *pyk* deletion
pyk ver rev	TTCGAGGGCGGTCAACATAGAGC	Verification of *pyk* deletion
pheA fw	GCCTGCAGGTCGACTCTAGAGGAAAGGAGGCCCTTCAGATGACATCGGAAAACCCGTTACTGG	Amplification of *pheA*
pheA rv	AACGACGGCCAGTGAATTCGAGCTCTCAGGTTGGATCAACAGGCACTACG	Amplification of *pheA*
aroK fw	GTTGATCCAACCTGACAGAGACAACAGCTCTACTAGGCAGTAATATCGAAAGGAGGTTTTTTATGGAGGGCAAAGCGTAT	Amplification of *aroK*
aroK rev	CGAGCTCGGTACCCGGGGATCTTAGTAGATTGAAGCTCCGTCG	Amplification of *aroK*
dpkA-pVW-fw	GCCAAGCTTGCATGCCTGCACAAGCGCACAAATCGAGGTCGAAAAGGA GGTTTTTTATGTCCGCACCTTCCACCAG	Amplification of *dpkA*
dpkA-pVW-rv	GGGATCCTCTAGAGTCGACCTGCATCAGCCAAGCAGCTCTTTCA	Amplification of *dpkA*
pheA fw	GCCAAGCTTGCATGCCTGCAGAAAGGAGGCCCTTCAGATGACATCGGAAAACCCGTTACTGG	Amplification of *pheA*
pheA rev	AACGACGGCCAGTGAATTCGAGCTCTCAGGTTGGATCAACAGGCACTACG	Amplification of *pheA*
pheA T326P fw	CACAATCTGATTATGCCCCGTCTGGAATCAC	Introduction of point mutation
pheA T326P rev	GTGATTCCAGACGGGGCATAATCAGATTGTG	Introduction of point mutation
pEC-XT99A-psyn-fw	TCAGTGAGCGAGGAAGC	Verification of pEC-XT99A transformants
pEC-XT99A-rev	TACTGCCGCCAGGCAAATTC	Verification of pEC-XT99A transformants

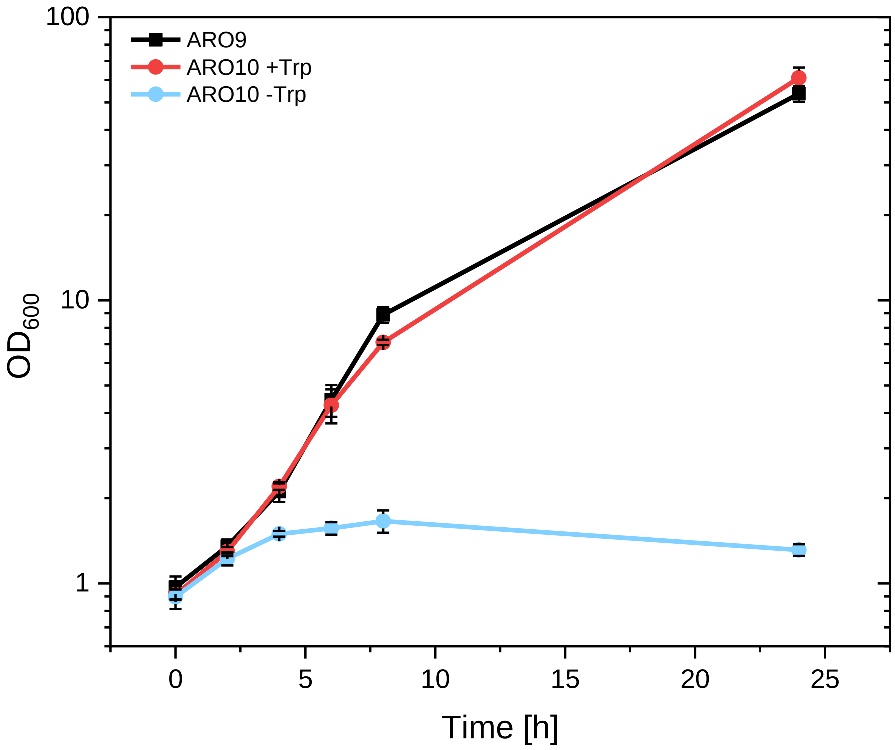

Figure S1: Verification of tryptophan auxotrophy of ARO10. *C. glutamicum* strain ARO10 was cultivated in minimal medium with supplementation of 0.8 mM tryptophan (circle red) or without tryptophan (circle blue) for 24 h. ARO9 was chosen as a control (square black).

After deletion of the anthranilate synthase (*trpEG*) in ARO9, the resulting strain ARO10 became auxotrophic for tryptophan, thus supplementation of the aromatic amino acid tryptophane is required (Figure S1).

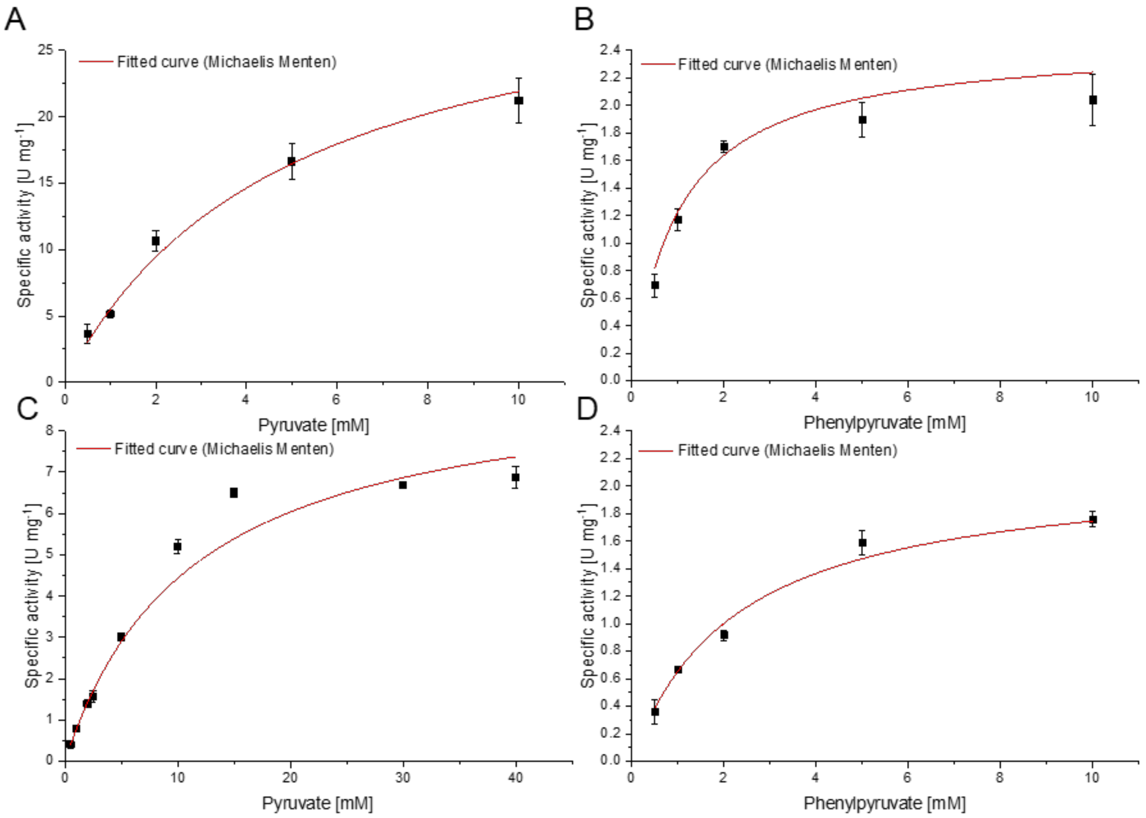

Figure S2: Michaelis-Menten kinetics for DpkA and DpkA^{P262A,M141L} with pyruvate and phenylpyruvate. Determination of K_m of DpkA WT for pyruvate (A) and phenylpyruvate (B) with similar MMA concentration and determination of K_m of DpkA^{P262A,M141L} for pyruvate (C) and phenylpyruvate (D) are depicted. K_m values were calculated using Origin with the function "Enzyme kinetics". Values of technical triplicates are shown.

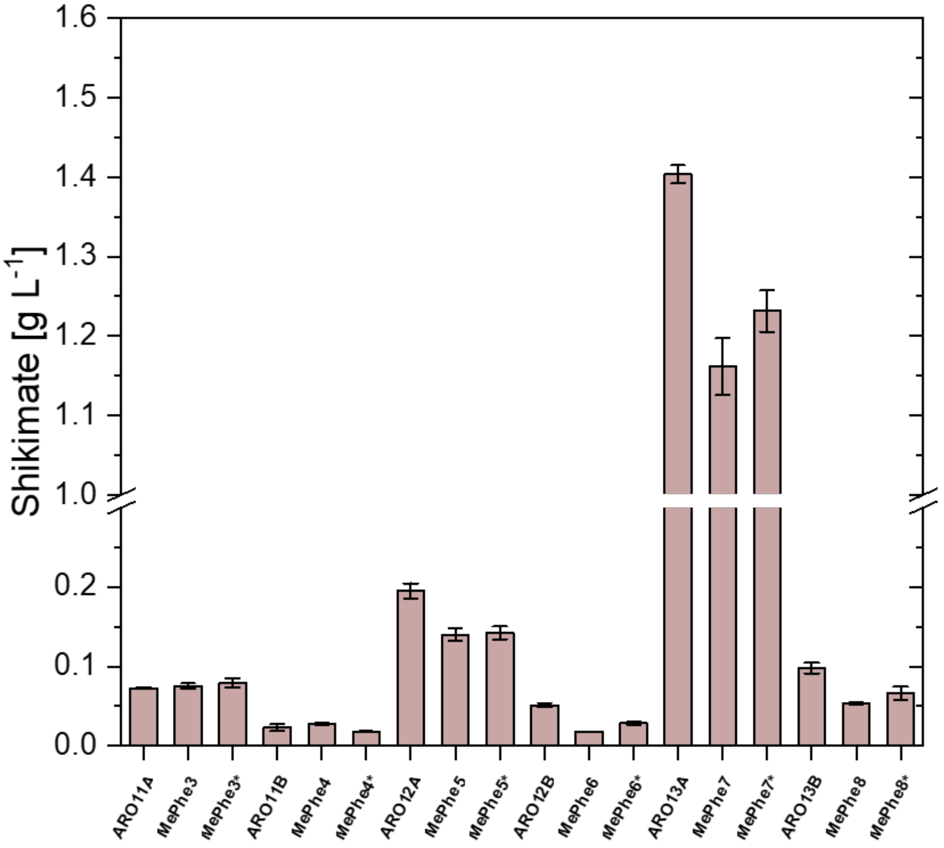

Figure S3: Production of shikimate by *C. glutamicum* ARO and MePhe strains. The strains were grown in Duetz-plates in CGXII medium containing 50% nitrogen and 20 g L^{-1} glucose as sole carbon source for 72 h. Means and standard deviations of technical triplicates are shown.

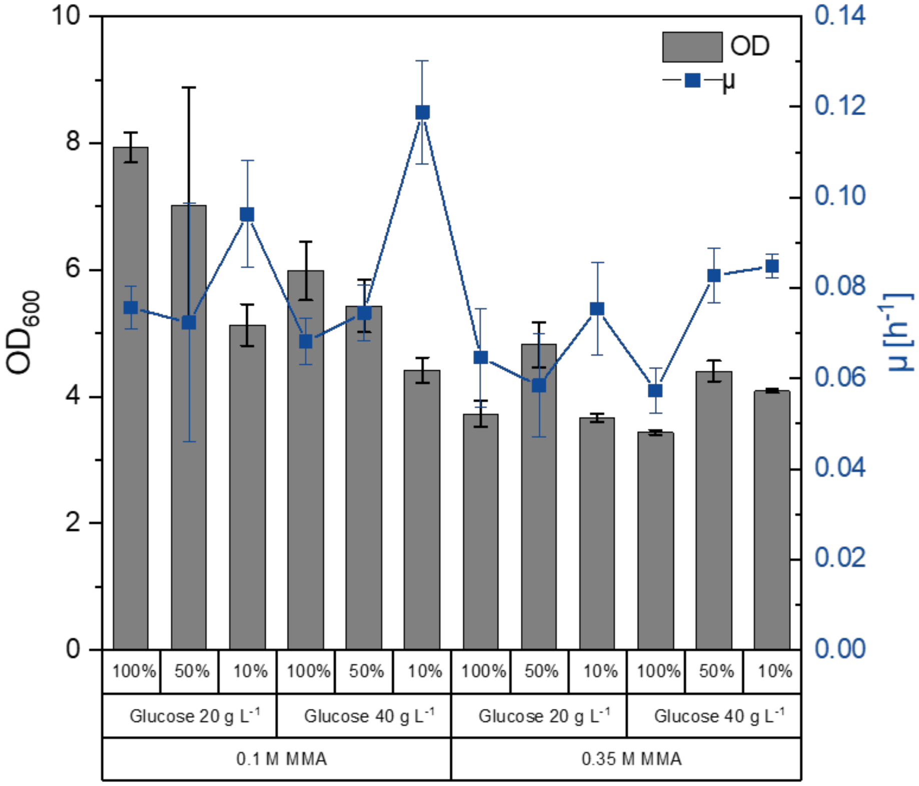

Figure S4: Growth of *C. glutamicum* strain NMePhe5* with different culture media compositions. StrainNMePhe5* was grown for 72h in Duetz-plates using CGXII media with the indicated concentrations of alkylamine donor (0.1 M and 0.35 M MMA), carbon source (20 g L^{-1} and 40 g L^{-1} glucose), and nitrogen source (10%, 50% and 100% of the concentrations of the nitrogen sources urea and ammonium sulfate). Means and standard deviations from triplicate cultures are depicted.

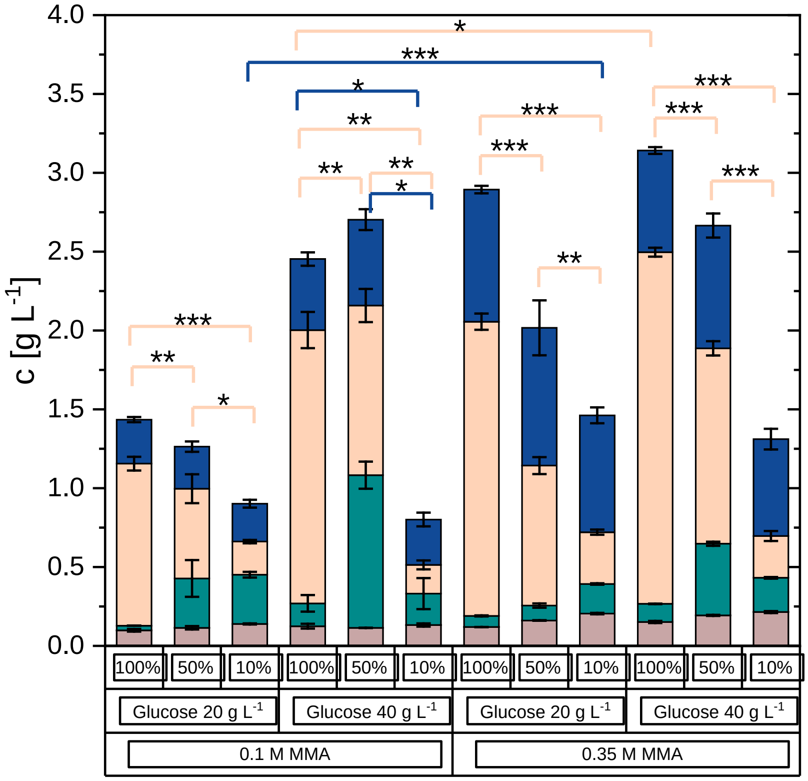

Figure S 5 Production of NMePhe (blue), NMeAla (orange), phenylpyruvate (cyan), and shikimate (light brown) *by C. glutamicum* strain NMePhe5* with different culture media compositions. Strain NMePhe5* was grown using CGXII media with the indicated concentrations of alkylamine donor (0.1 M and 0.35 M MMA), carbon source (20 g L^{-1} and 40 g L^{-1} glucose), and nitrogen source (10%, 50% and 100% of the concentrations of the nitrogen sources urea and ammonium sulfate). Means and standard deviations of triplicate cultures are depicted. Significance has been determined for NMeAla (orange) and NMePhe (blue) concentrations based on a two-sided unpaired Welch-t-test (*: $p \leq 0.05$, **: $p \leq 0.01$, ***: $p \leq 0.001$). When not indicated, no significant difference in NMePhe production was detected by comparing constant MMA and glucose concentration.
